# Bioinformatic Description of Immunotherapy Targets for Pediatric T-Cell Leukemia and the Impact of Normal Gene Sets Used for Comparison

**DOI:** 10.3389/fonc.2014.00134

**Published:** 2014-06-10

**Authors:** Rimas J. Orentas, Jessica Nordlund, Jianbin He, Sivasish Sindiri, Crystal Mackall, Terry J. Fry, Javed Khan

**Affiliations:** ^1^Pediatric Oncology Branch, Center for Cancer Research, National Cancer Institute, National Institutes of Health, Bethesda, MD, USA; ^2^Molecular Medicine, Department of Medical Sciences and Science for Life Laboratory, Uppsala University, Uppsala, Sweden

**Keywords:** immunotherapy, chimeric antigen receptors, antibody therapy, adoptive immunotherapy, pediatric leukemia, T-ALL, TALLA-1, HHIP

## Abstract

Pediatric lymphoid leukemia has the highest cure rate of all pediatric malignancies, yet due to its prevalence, still accounts for the majority of childhood cancer deaths and requires long-term highly toxic therapy. The ability to target B-cell ALL with immunoglobulin-like binders, whether anti-CD22 antibody or anti-CD19 CAR-Ts, has impacted treatment options for some patients. The development of new ways to target B-cell antigens continues at rapid pace. T-cell ALL accounts for up to 20% of childhood leukemia but has yet to see a set of high-value immunotherapeutic targets identified. To find new targets for T-ALL immunotherapy, we employed a bioinformatic comparison to broad normal tissue arrays, hematopoietic stem cells (HSC), and mature lymphocytes, then filtered the results for transcripts encoding plasma membrane proteins. T-ALL bears a core T-cell signature and transcripts encoding TCR/CD3 components and canonical markers of T-cell development predominate, especially when comparison was made to normal tissue or HSC. However, when comparison to mature lymphocytes was also undertaken, we identified two antigens that may drive, or be associated with leukemogenesis; TALLA-1 and hedgehog interacting protein. In addition, TCR subfamilies, CD1, activation and adhesion markers, membrane-organizing molecules, and receptors linked to metabolism and inflammation were also identified. Of these, only CD52, CD37, and CD98 are currently being targeted clinically. This work provides a set of targets to be considered for future development of immunotherapies for T-ALL.

## Introduction

The origin of leukemia may be best understood through the specific genomic mutations that alter the regulation, growth, and differentiation of the lymphocytic cells that comprise the disease. These mutations both define the biology of the disease at presentation and can also reveal the genetic origins of the leukemic stem cell. The promise of next-generation sequencing technologies to more fully describe these mutations is starting to be fulfilled as exomic or genomic sequencing has begun to supplement and may someday replace traditional methods of leukemia diagnosis and classification based on pathology, immunophenotyping, and molecular cytogenetics (including FISH, fluorescence-based *in situ* hybridization, and PCR, polymerase chain reaction, for known genetic lesions) (1).

Genomic technology, however, cannot stand on its own as verification of target expression is still required at the protein level. Thus, it is immunophenotyping that ultimately informs the field of immunotherapeutics whether or not a genetic target could serve as therapeutic target for either antibody or T-cells transduced to express chimeric antigen receptors (CAR-Ts). The advent of CD19–CAR-T-cell therapy has impacted the treatment of pre-B-cell ALL for some patients with advanced disease. Indeed, we and others have proposed a number of targets that may be suitable for pediatric B-ALL (2, 3). However, attractive targets for T-cell leukemia have yet to be recognized and exploited. We present here potential targets for treating T-cell ALL with antibody or CAR-Ts, using strategies developed for the analysis of pediatric solid tumors and B-ALL (4).

In 1993, Pui et al. reviewed ontogeny marker expression in T-ALL in light of normal T-cell antigen expression during thymic development (5). T-ALL was considered as either prothymocyte- (expressing CD7), early thymocyte- (expressing CD5, CD2, and CD1), intermediate thymocyte- (CD1, CD4, or CD8), or mature thymocyte-like (CD3 and TCR surface expressed). The CD1 antigen, expressed on cortical thymocytes, Langerhans cells, and a subset of B-cells, is the only one of these developmental antigens to be turned off upon reaching T-cell maturity. Reinherz originally proposed that T-ALL be classified along the lines of CD1 and CD3 expression with stage I (early thymocyte) expressing CD2, CD5, CD7, and no CD1, CD4, CD8, or CD3; stage 2 (intermediate) expressing CD1, CD2, CD5, and CD7 with variable 4 and 8, and weak CD3; and stage 3 (mature) expressing CD2, CD3, CD5, CD7, and CD4 or CD8 (usually only one or the other) (6, 7). In most simplistic terms, mature or medullary T-ALL expresses surface CD3, but not CD1a. Cortical or thymic T-ALL expresses CD1a, but not surface CD3; and early T-cell precursor T-ALL (ETP-ALL, which encompasses Pro-T-ALL and Pre-T-ALL) does not express CD3 or CD1a.

The answer to the challenge of finding T-cell restricted targets (that is a mature T-cell antigen present on ALL that can be safely eliminated, as CD19 for B-cells), or a more T-ALL restricted target (especially for the more immature forms of the disease) may lay in the nature of the progenitor cell itself. As elegantly presented by the St. Jude – Washington University Pediatric Cancer Genome Project, early precursor T-cell ALL shares many similarities to double negative thymocytes that have the potential to differentiate into cells of either T-cell or myeloid lineage (8, 9). This pluripotency makes the antigenic expression profile for T-ALL far more generalized. At the other end of the spectrum, the most mature forms of T-ALL may benefit from new immunotherapeutic approaches that target the T-cell receptor, specifically, different subclasses that have clonally expanded. Although this was once deemed an approach to be of little interest due to the low number of cases, and the need for an almost individualized treatment approach, the success of CAR-T-cell therapy, which is the essence of personalized or individualized medicine, has brought this approach to the fore once more.

## Data Interrogation and Results

T-cell ALL (acute lymphocyte/lymphoblastic leukemia) accounts for 15–18% of all childhood leukemias and 25% of ALL in adults (5, 10). However, we have yet to see a set of high-value targets proposed for T-cell ALL as we have for B- or pre-B-ALL. To that end we undertook a bioinformatics approach to describe the cell surface proteins expressed on T-ALL. Although we have begun to accumulate and analyze T-ALL cases at the Pediatric Oncology Branch of the NCI, a large data set was recently made available at Gene Expression Omnibus (GEO) using the Affymetrix Human Genome U133 Plus 2.0 platform. In this submission, GSE47051, over 100 cases of pediatric T-ALL and pre-B-ALL are available for analysis. The samples were of high quality and derived from bone marrow or peripheral blood aspirates of pediatric ALL patients enrolled in two co-operative group trials (NOPHO, Nordic Society for Pediatric Hematology-Oncology), fully described in the original publications for this sample set (11, 12). Samples were obtained under informed consent, approved by the Regional Ethical Board in Uppsala, Sweden, and in accordance with the guidelines set forth in the Declaration of Helsinki.

**Figure 1:**
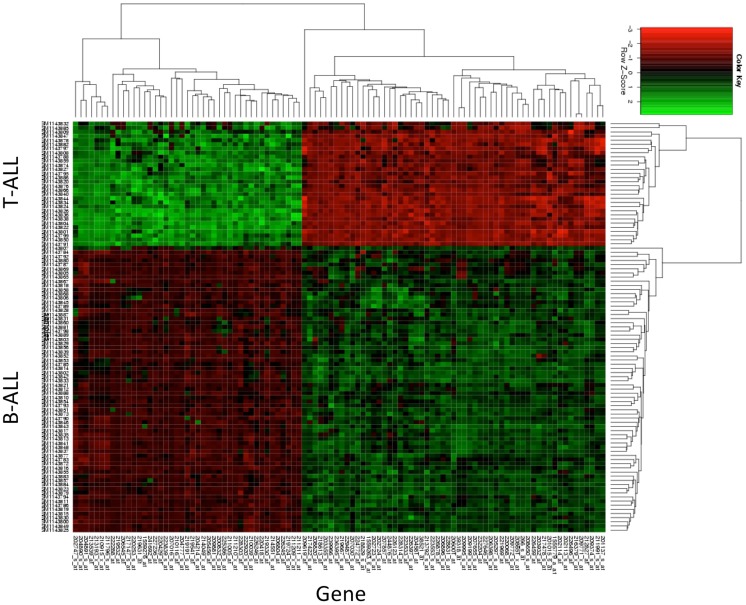
**Hierarchical clustering of T-ALL and pre-B-ALL**. Samples from GEO data set GSE47051 were downloaded and then clustered without reference to their diagnostic category (T-ALL or B-ALL). The top genes driving the subsequent clustering are listed in the *x*-axis, and the disease categories of those samples on the *y*-axis. Gene expression (log_2_cs) was normalized by *z* score (*x*-mean/std.dev.) across all leukemia samples for purposes of comparison.

**Figure 2:**
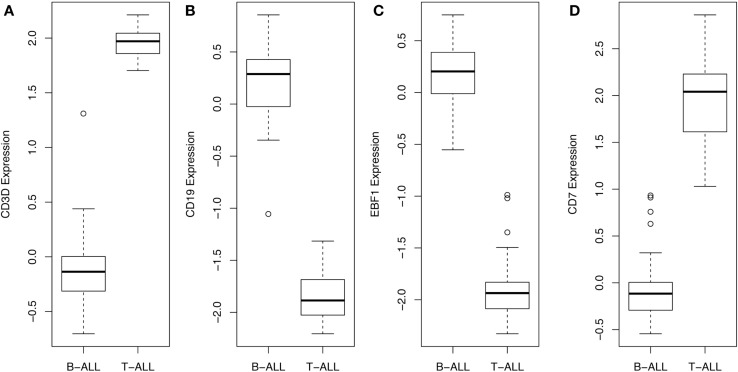
**Gene expression values, across all samples, for top scoring hits**. The average and range of values of the top four genes are presented in this box and whisker plot, demonstrating that CD3D and CD7 for T-ALL and CD19 and EBF1 for BCP-ALL (B-ALL) can be used to readily distinguish and verify the original pathological classification of these disease samples.

By navigating to the GEO home page, the CEL files for these annotated samples were accessed and analyzed. To test the accuracy of the pathological categorization of this sample set, we carried out hierarchical clustering using the freely available ArrayMiningtool (Online Microarray Data Mining Tool)^1^ (13). In Figure 1 we demonstrated the partitioning of the pre-B-ALL and T-ALL samples, confirming their original pathological classification. Moreover, the genes driving this classification are unmistakably well-recognized components of ALL, Table 1. The T-ALL set contains CD3D, CD7, and CD28; while in the pre-B-ALL set we find CD19, CD79B, and CD22. In Table 1 we present the top 10 cell surface genes with regard to Pearson correlation (gene vs. outcome), and in Figure 2, we present the top four overall scores, as expressed by their respective sample sets. Not surprisingly, the gene expression values that drove this classification were highly divergent from each other, such as CD3D for T-ALL and CD19 and EBF1 (early B-cell factor 1) for pre-B-ALL. Having confidence in the submitted sample classification, we then analyzed the T-ALL and pre-B-ALL samples for differences in average gene expression in comparison to broad normal tissue arrays. For each cell surface membrane transcript, an average expression level was calculated, and then compared to the average expression of the transcript in normal tissue, thus defining a potential space for immunotherapeutic intervention. Methods for this analysis were published previously (4). To confirm our analysis, we also analyzed normal and disease gene expression files using the Partek^®^ Genomics Suite™ v6.5. The GEO database was used to download individual CEL files into the software suite, and data analyzed for quality, differential expression, and with statistical packages for differential gene expression (14–16).

**Table 1:** **Top 10 cell surface genes driving classification**.

Identifier	Gene name	Description	PC	Fold-change
**SURFACE T-ALL MARKERS**				
213539_at	CD3D	CD3d molecule, delta chain of TCR/CD3	0.97	53.6
214551_s_at	CD7	CD7 molecule	0.94	17.95
205456_at	CD3E	CD3e molecule, epsilon chain of TCR/CD3	0.93	17.52
226246_at	KCTD1	K channel tetramerization domain containing 1	0.92	7.87
211796_s_at	TRBC2	T-cell receptor beta constant 2	0.91	61.21
226245_at	KCTD1	K channel tetramerization domain containing 1	0.91	10.55
206545_at	CD28	CD28 molecule	0.91	27.11
227236_at	TSPAN2	Tetraspanin 2	0.9	10.94
217147_s_at	TRAT1	TCR-associated transmembrane adaptor 1	0.89	28.59
220253_s_at	LRP12	LDLR-related protein 12	0.88	9.55
**SURFACE B-ALL MARKERS**				
206398_s_at	CD19	CD19 molecule	−0.96	0.03
226878_at	HLA-DOA	MHC class II, DO alpha	−0.92	0.07
205297_s_at	CD79B	CD79b molecule, Ig-associated beta	−0.9	0.07
228831_s_at	GNG7	G-protein, gamma 7	−0.9	0.07
209374_s_at	IGHM	Immunoglobulin heavy constant mu	−0.89	0.06
223533_at	LRRC8C	Leucine-rich repeat containing 8 family, C	−0.89	0.11
220068_at	VPREB3	Pre-B lymphocyte 3	−0.89	0.02
38521_at	CD22	CD22 molecule	−0.88	0.24
207857_at	LILRA2	Leukocyte ig-like R, subfam. A (w/TM domain), 2	−0.87	0.06
217478_s_at	HLA-DMA	MHC, class II, DM alpha	−0.87	0.05

*Genes were clustered, and then ordered according to Pearson correlation co-efficient (PC, gene vs. outcome). The genes scoring as a positive (T-ALL markers) or negative (B-ALL markers), confirm the original designation in GEO*.

Table 2 lists the top 40 genes for each disease category, as a function of the ratio of an individual transcript’s expression level in ALL vs. a set of 117 normal tissues, our broad normal tissue array. A third of the transcripts (15 of the 46 transcripts, 33%) were present in both pre-B-ALL and T-ALL. These transcripts are by definition shared more between the two leukemias than with normal tissue. They are (bold italics in the table): PTGER4, ITGA4, CD37, CD52, CD62L (L-selectin), CXCR4, CD69, EVI2B (CD361), SLC39A8, MICB, LRRC70, CLELC2B, HMHA1, LST1, and CMTM6 (CKLFSF6).

**Table 2:** **T-ALL and B-ALL cell surface immunotherapy targets**.

T-ALL vs. normal tissue					B-ALL vs. normal tissue				
Rank (T)	Fold-change	Symbol	Comment	Affy ID	Rank (T)	Fold-change	Symbol	Comment	Affy ID
1a	97.6	TCRBV5-4	TCR beta chain	211796_s_at	1	90.8	***CD69***		209795_at
2	67.5	CD3D	CD3-delta	213539_at	2	83.3	VPREB1	Pre-B lymphocyte 1	221349_at
3	64.9	***PTGER4***	Prostaglandin E, R4	204897_at	3	57.3	***CD52***		34210_at
4	60.8	***CD69***		209795_at	4	53.9	IGHM	Ig HC-M	212827_at
1b	56.7	TRBC1	TCR beta chain	210915_x_at	5	44.3	***CXCR4***		217028_at
1c	48.9	TCBC1/BV19	TCRB	213193_x_at	6	43.1	IGGL1	Surrogate light chain	206660_at
5	39.4	***ITGA4***	CD49d	213416_at	7	39.7	CD24		208650_s_at
6	36.7	***CD52***		34210_at	8	35.4	VPREB3		220068_at
7	35.3	CD1E		215784_at	9	34.9	LILRA2		207857_at
1d	28.6	TCRDC	TCR delta constant	216191_s_at	10	27.7	***EVI2B***		211742_s_at
8	26.1	***SELL***	CD62L/selectin L	204563_at	11	26.9	MME	CD10/CALLA	203434_s_at
9	25.6	CD28		206545_at	12	26.7	***PTGER4***		204897_at
10	24.6	***CXCR4***		217028_at	13	23.4	***ITGA4***		213416_at
11	23.1	***EVI2B***	CD361	211742_s_at	14	22.0	CD79A		205049_s_at
12	20.9	***SLC39A8***	Zinc transporter	209267_s_at	15	21.7	CD79B		205297_s_at
13	20.2	***ICAM-3/CD50***		204949_at	16	18.1	***CLEC2B***		209732_at
14	19.6	PTPRC	CD45R	212588_at	17	16.9	***SLC39A8***		209267_s_at
15	17.1	CD96		206761_at	18	16.7	CD19		206398_s_at
16	16.4	CD7		214551_s_at	19a	16.7	HLA-DRA	MHC class II	208894_at
17	15.3	ITGAL/CD11a		1554240_a_at	20	16.7	***MICB***		206247_at
18	14.4	***MICB***	Ligand for NKG2D	206247_at	21	14.7	***LRRC70***		238488_at
19	13.0	***LRRC70***	SLRN	238488_at	19b	14.2	HLA-DQ1		212671_s_at
20	13.0	CD2		205831_at	22	14.2	PAG1		225622_at
21	12.6	CD3E	CD3-epsilon	205456_at	23	13.0	LILRB2		210146_x_at
1e	12.4	TCRG2	TCR gamma C 2	213060_s_at	24	12.8	CD58		211744_s_at
22	12.2	***CLEC2B***	C-type lectin	209732_at	25	12.3	***CD37***		204192_at
23	11.6	CD247	TCR zeta chain	210031_at	26	12.3	HMHB1		208302_at
24	11.4	IL2RG	CD132	204116_at	27	11.1	FLT3		206674_at
1f	11.2	TCR delta		213830_at	28	10.4	CD72		215925_s_at
25	11.0	CD1B		206749_at	29	10.3	CD53		203416_at
26	11.0	CD3G	CD3 gamma	206804_at	30	10.2	LRR8C		223533_at
27	10.6	P2RY8		229686_at	19c	10.1	HLA-DPA1		211991_s_at
28	10.4	HMMR	RHAMM/CD168	207165_at	31	9.8	FAIM3	FcR for IgM	221601_s_at
29	10.2	***CD37***		204192_at	32	9.7	CMTM7	CKLFSF7	226017_at
30	10.2	CD99		201028_s_at	33	9.6	TLR1	CD281	210176_at
31	10.2	HHIP		1556037_s_at	34	9.5	MS4A1		228592_at
32	8.5	IL-7R		226218_at	35	9.1	***LST1***		214181_x_at
33	8.5	***LST1***		214181_x_at	19d	9.1	HLA-DMA		217478_s_at
34	8.3	PTPRCAP	CD45AP	204960_at	36	8.7	IGHD		213674_x_at
35	7.8	***HMHA1***	Minor HA-1	212873_at	37	8.4	***CMTM6***		217947_at
36	7.5	LPAR6	Lysophosphatidic acid R	218589_at	38	8.3	CD97		237510_at
37	7.1	ORAI2	Ca++ channel modulator	217529_at	39	8.1	***MHMA1***		212873_at
1g	7.0	TCRGC 2		211144_x_at	40	7.9	MILR1		217513_at
38	6.9	CD84		244352_at	41	7.9	***ICAM-3***		204949_at
39	6.8	TSHR		215442_s_at	42	7.7	***SELL***		204563_at
40	6.8	***CMTM6***	CKLFSF6	217947_at	43	7.5	LY9	CD229	231124_x_at

*The left hand side of the table lists the top scoring hits for T-ALL, and the right side of the table shows the top hits for pre-B-ALL, expressed as the ratio of expression in the leukemia over the expression of that transcript in normal tissue (117 samples covering all major tissue types of the body). These values were calculated using the ANOVA module in PartekGenomics Suite (Partek, Inc., St. Louis, MO, USA), where *T* tests, ratio of average expression, and the associated *p* values were calculated. Rank, indicates the numerical order list, with the TCR subunits for T-ALL and the MHC class II genes for B-ALL marked with a letter so as to indicate the presence of very similar hits. The affy ID generating the highest scoring hit is reported, and hits for identical genes were not repeated. The comment column is used to report alternate nomenclature. Boxes are used to indicate genes we have previously described as over-expressed in B-ALL (4). Gene symbols in bold italic are present in both T-ALL and B-ALL lists. The highest *p* value for all the analyses presented in this table was 2.2 × 10^−30^, and thus *p* values are not presented in the table*.

### Shared pre-B-ALL and T-ALL genes

*SLC39A8* is the easiest to classify as this zinc transporter is a solute carrier protein expressed during inflammation. In short, it is a response to metabolic demand (17). The next group of markers that naturally fall together has to do with core homing and adhesion functions of lymphocytes, specifically *CD37*, a tetraspanin that associates with integrins; *CD49d*, an integrin expressed on activated lymphocytes; *CD62L*, a homing receptor; *CD52*, an adhesion receptor; *CXCR4*, whose natural ligand is SDF-1 and mediates homing to the bone marrow. Tetraspanins organize plasma membrane domains in order to co-ordinate signaling and cellular functions. In B-cells, the CD81/CD21/CD19 co-receptor complex interacts with CD82 and lipid rafts, while in T-cells the CD151/CD81/CD37/Tssc-6 complex is thought to perform a similar structural-signaling role (18, 19). This linkage to the tetraspanin *CD37* is significant, as our data now link the expression of CD37 with known adhesion receptors of the integrin family in ALL. Anti-CD37 antibody is currently being evaluated in adult B-cell malignancies (NHL, CLL, MM, ALL) as a drug conjugate with the maytansine derivative DM1 (20).

*CXCR4* is over-expressed in more than 20 different cancers, and expression in normal tissues (other than in the CD34 stem cell niche in bone marrow) is measurably lower (21–23). *CD49d* (alpha 4, beta 1/beta 7 integrin) is an adhesion receptor that mediates both rolling and firm adhesion to endothelium (24). CD49D expression in tandem with CXCR4 has been studied in pediatric and adult ALL. In children with ALL, no difference in expression levels was correlated to outcome, while adults with high CXCR4 and low CD49D (VLA-4) did worse (25).

*CD62L* is the canonical homing receptor for lymphocytes to enter secondary lymphoid tissues via high endothelial venules. Recently, targeting CD62L has been proposed for CLL, and thus it may be a viable target in pediatric ALL (26). *CD52* is the well-characterized target of the anti-CAMPATH-1 antibody alemtuzumab, and is expressed on activated T-cells. This represents a second opportunity to more fully explore in pediatric ALL. *CLEC2B* (C-type lectin domain family 2B), is encoded in the NK cell killer gene complex on chromosome 12, and is also known as activation induced C-type lectin of T-cells, called AICL (27). AICL/CLELC2B map adjacent to CD69 whose C-type lectin domain is similar in sequence. *CD69*, a well-described activation marker for T-cells has also been found to be valid marker for prognosis in CLL, reflecting its ability to be up-regulated in either cell lineage (28). *MICB* (MHC class I polypeptide-related sequence B), is a ligand for NKG2D, and it thus activates T or NK cells. The shedding of the MICB protein is thought to help tumors evade NK-mediated immunosurveillance (29). Although not expressed on pre-B-ALL, *CD96* is also an immunoglobulin superfamily member, and we discuss it here because it is also a ligand for activated T or NK cells. CD96 has also been described as a leukemia stem cell marker in AML (30). Since the expression of CD96 is low or absent on normal hematopoietic stem cells (HSC), Staudinger et al., have proposed using anti-CD96 antibody to purge bone marrow during hematopoietic stem cell transplantation (HSCT) for the disease (31).

*CD361* (*EVI2B*) is a recently described antigen that was classified in 10th human leukocyte differentiation antigens (HLDA) conference as part of the B-cell panel, showing specific staining of ALL (32). This gene is found in an intron of the NF1 (neurofibromatosis type 1) gene, along with EVI2A and OMpg, being transcribed in the opposite direction from NF1 (33). Little is known of its function, and it may be over-expressed simply because of its genomic location. The *CMTM6* (CKLF-like MARVEL transmembrane containing 6, CKLFSF6) is a chemokine-like gene that also contains a domain that bears sequence similar to tetraspanins. Thus, it appears to play both a signaling and a membrane-organizing role. The biological function is still being investigated.

*LRRC70* (leucine-rich repeat containing 70/synleurin), is also not well-described and may play a role in cell adhesion or may bind a growth factor (34). Few functional studies have been carried out on the LRR protein family. *PTGER4*, is a prostaglandin receptor. This G-protein-coupled receptor is known to activate T-cells. Lymphocytic colitis was recently shown to be associated with increased TNFA, IFNG, and PTGER4, and a role for prostaglandin proposed in activating pathogenic lymphocytes (35). *LST1* (leukocyte specific transcript 1) is expressed normally in cells of myeloid lineage, serving as transmembrane adaptor protein, interacting with the SHP-1 and SHP-2 phosphatases (36). There is no described association with LST1 and ALL to this point.

Mutis et al., generated cytotoxic T-cells restricted to the non-self HLA molecule HA-1/*MMHA1* (histocompatibility, minor, HA-1), and proposed using this reactivity to actively target residual disease during HSCT (37, 38). The restriction of this antigen to the hematopoietic system highlights the fact that HSCT, which is often part of ALL therapy, could be augmented by targeting leukemia-expressed HA-1. In clinical trials, HA-1 and HA-2 peptide vaccines are being tested for the induction of increased graft vs. leukemia effect following allogeneic HSCT (NCT00943293). In reviewing this list of genes over-expressed in both T-ALL and pre-B-ALL, in comparison to normal tissue, a general picture of immune activation arises. While some of the proteins encoded by the transcripts expressed by both T-ALL and B-ALL are not as well-characterized, they still fit this theme and are also likely to be expressed throughout the immune system. Obvious therapeutic correlation can be made for CD52 and CD37. Antibody to CD96 is used for purging marrow, because it is too broadly expressed in normal tissue to be targeted by an active agent. The ability of MICB and CD96 to activate T-cells or NK may also be an important insight, as adoptive immunotherapy with T-cells engineered to express NK ligands has been demonstrated and their presence may therefore be associated with an activated mature phenotype (39). Also of importance to immunotherapy is the presence of minor antigen (HA-1) in ALL that may be exploited during HSCT. We now turn our attention to the transcripts expressed in the disease most in need of new approaches, pediatric T-ALL.

### T-ALL genes

Among the T-ALL transcripts listed as being over-expressed in Table 2, 7 of 46 are *TCR chains* (TCRBV5-4, TCRBC1, TCBC1/TCRBV, TCRDC, TCRG2, TCR delta, and TCRGC2) and 4 are members of the *CD3 complex* (CD3-delta, -epsilon, -gamma, and -zeta), making 11 of 46 (23%) of the T-ALL hits in Table 2 directly related to clonotypic T-cell marker expression. This analysis does not inform us directly if these molecules are on the surface, as the presence of the surface protein CD3-epsilon is the key diagnostic criterion for classification of T-ALL as being mature/medullary type, but many of these are re-arranged TCR chains indicating the T-ALL data set we are analyzing is weighted toward a more mature phenotype. Thinking of these re-arranged proteins as immunotherapeutic targets will be discussed later.

Transcripts for many canonical normal T-cell markers are also seen in T-ALL: such as, CD2, CD7, CD11a, CD28, CD45R, CD45AP, CD84, CD99, IL2RG, IL-7R, and the CD1 family markers CD1E and CD1B. If we consider this set of transcripts as an aggregate diagnostic of a single case, the presence of *CD2* and *CD1* transcripts, along with *CD7* would classify this sample as a cortical or thymic T-ALL, which is in keeping with the predominance of re-arranged TCR transcripts detected (as opposed to an early T-cell precursor type ALL). *CD84* is in the SLAM (signaling lymphocyte activation molecule) subset of the CD2 family of proteins, and mediates T-cell activation through homotypic adhesion (40). *CD28* is the well-characterized second signaling molecule, *CD11A* (LFA-1 alpha chain) is a well-recognized lymphocyte integrin, and *CD45* is LCA (leukocyte common antigen). *CD45AP* is known to positively regulate CD45 function. The transcripts for *IL2* and *IL7 receptor chains* are also in keeping with the T-cell transcriptome of T-ALL. *CD99* is a well-characterized marker of T-ALL and in pediatric disease its high level of expression has been proposed as a marker for detecting minimal residual disease along with CD3, CD7, and/or TdT (41). Hence, this set of transcripts is largely a picture of a normal activated immune cell.

The expression of CD1A is a key diagnostic for T-ALL, representing the key transition, along with high expression of CD2, to a more mature phenotype. In our analysis, we detected transcripts for *CD1B* and *CD1E*. CD1A is thought to be the primary cell surface CD1 family member while CD1B interacts with intracellular lipids and CD1E functions as a lipid chaperone, reviewed in Ref. (42). Nevertheless, CD1B clearly presents mycobacterial antigens to T-cells and is on the surface (43). In studying LEF1 mutations in pediatric T-ALL, Gutierrez et al. demonstrated that this subset of pediatric T-ALL was arrested at the cortical stage and they demonstrated the presence of CD1B and CD1E by gene expression profiling (44). We can infer from this that the CD1 family is induced as a group, and also hope that unique subsets of lipid antigens may be present on T-ALL. None have yet been reported.

Three immune cell adhesion receptors are over-expressed in T-ALL. *ICAM-3* (CD50) is constitutively expressed on most B-cell malignancies, except for Hodgkin lymphoma, and myeloid cells, but appears to be absent from other tissues, with the possible exception of tumor-associated vasculature (45, 46). We report here that it is likely to be found on pediatric T-ALL as well. *HMMR* (hyaluronan-mediated motility receptor, RHAMM, or CD168), is found in a complex containing BRCA1 in breast tissue where it may govern apicobasal polarity (47). TCRs recognizing RHAMM were generated in a model system and found to inhibit AML growth in a xenogeneic system (48). However, these T-cells also recognized CD34+ HSC in an HLA-A2 restricted manner. Thus, an active T-cell population targeting HMMR could only be envisioned for clinical use in an HLA mismatch setting.

*LPAR6*, lysophosphatidic acid receptor 6, is another interesting transcript that may be expressed either for its contribution to cellular transformation or it may be part of an active genomic locus, as it is transcribed from an intron of the RB gene (49). On its own, this G-coupled receptor is known to stimulate cell activation upon encountering its ligand, lysophosphatidic acid, and may play a role in tumor cell tissue invasion (50). Like the zinc transporter, another T-cell metabolic protein, *ORAI2* (ORAI calcium release-activated calcium modulator 2) is over-expressed in T-ALL. It is likely to function in a manner similar to ORAI1 where STIM1 activates the translocation of this calcium channel to the surface of the T-cell upon depletion of intracellular calcium stores (51).

Three transcripts expressed by T-ALL are a little more enigmatic, but reflect the fact that we are dealing with a transformed cell population. *HHIP* (Hedgehog interacting protein) interacts with all forms of sonic hedgehog (SHH), a secreted factor that guides tissue formation during development, and is a key mediator of its function (52). HHIP function on T-ALL has not been explored, but an AML study has described the ability of AML-derived stromal cells to express HHIP and to interact with AML stem cells that now re-express SHH and its receptor, smoothened (SMO) (53). *TSHR* (thyroid stimulating hormone receptor) is not normally considered a T-cell antigen, but mutations of this receptor have been associated with increased vasculature and the development of pituitary adenomas (54, 55). Clinical studies with TSHR primarily focus on Grave’s disease. *P2RY8* (purinergic receptor P2Y, G-protein-coupled 8), is best known as supplying the promoter that drives CRLF/TSLPR overexpression, upon deletion of an intervening pseudoautosomal region, in certain cases of B-ALL (56, 57). In a fascinating study, Fujiwara et al., screened for transforming genes in the rare biphenotypic acute leukemia (BAL), which bears both lymphoid and myeloid markers (58). They found P2RY8 expression to activate a number of cellular activation pathways, including those mediated by CERB and Elk-1. Thus, P2RY8 may be over-expressed simply by residing in a very active genomic region or it may play a functional role in transforming leukemia cell clones.

In order to further analyze T-ALL gene expression signatures, we sought to determine if the normal gene sets we used for comparison had an impact on the type of transcripts identified as being over-expressed. We ran two subsequent analyses using CD34+ bone marrow-derived HSC, and normal peripheral blood mononuclear cells, as the normal tissue comparators, Table 3. HSC CEL files were submitted by Dehashis Sahoo to GEO as GSE32719 by the Weissman Lab at Stanford University, USA (59). We selected CD34+ HSC that was from normal donors aged 19–31 years old (*n* = 14). For normal PBMC, we used control patients from the GEO Submission GSE21942 by Kemppinen and Saarela at the University of Cambridge, UK (*n* = 15) (60). In the top half of Table 3, where comparison is made to HSC, a profile similar to the normal tissue array of Table 2 is seen, where T-cells transcripts predominate. In fact 60 and 70% of the transcripts (ranked by *T* score and fold-change, respectively), were shared. However, when comparison was made to PBMC, the percentage of shared transcripts dropped to 10 and 15% for *T* score and fold-change, respectively. Only three transcripts were shared between the lists featured in the top and bottom half (HSC vs. PBMC) Table 3 shows CD3D (the delta chain of the TCR complex), HHIP, and TALLA-1 (TSPAN7). The presence of T-cell associated like transcripts has been discussed, but HHIP and TALLA-1 warrant special attention.

**Table 3:** **Comparison of T-ALL to HSC and PBMC normal gene expression profiles**.

Rank	Symbol	Gene title	*T* score	Rank	Symbol	Gene title	Fold-change
**T-ALL VS. HSC**							
1	CD3D*	CD3d molecule, delta	50.7	1	CD3D*	CD3d molecule	140.1
2	CD3G*	CD3g molecule, gamma	24.2	2	TRBC1*	T-cell receptor beta constant 1	110.7
3	TRBC1*	TCR beta constant 1	22.1	3	CD1E*	CD1e molecule	54.6
4	CD99*	CD99 molecule	20.8	4	TRDC*	T-cell receptor delta constant	42.9
5	CD7*	CD7 molecule	20.1	5	TRDV3	T-cell receptor delta variable 3	39.4
6	B2M	Beta-2-microglobulin	18.6	6	CD2*	CD2 molecule	39.2
7	CD96*	CD96 molecule	17.2	7	CD28	CD28 molecule	31
8	IL2RG*	Interleukin 2 receptor, gamma	17.0	8	CD3E*	CD3e molecule, epsilon	28.6
9	CD3E*	CD3e molecule, epsilon	16.9	9	CD7*	CD7 molecule	26.6
10	LIME1	Lck interacting transmembrane adaptor 1	16.1	10	CD1B*	CD1b molecule	26
11	CD46	CD46 molecule	16.1	11	HHIP*	Hedgehog interacting protein	23.3
12	CD247*	CD247 molecule	16.0	12	CD96*	CD96 molecule	22.9
13	ITGB1	Integrin, beta 1, CD29	14.3	13	CD99*	CD99 molecule	22.1
14	LAX1	Lymphocyte transmembrane adaptor 1	13.5	14	CD3G*	CD3g molecule, gamma	21.2
15	ITGAE	Integrin, alpha E, CD103	13.4	15	AQP3	Aquaporin 3 (gill blood group)	20.7
16	HHIP*	Hedgehog interacting protein	12.9	16	IL-7R*	Interleukin 7 receptor	20.5
17	ITM2A	Integral membrane protein 2A	12.4	17	LPAR6	Lysophosphatidic acid receptor 6	18.5
18	CXCR4*	Chemokine (C-X-C motif) receptor 4	12.2	18	CD8A	CD8a molecule	18.5
19	CD28*	CD28 molecule	12.1	19	IL2RG*	Interleukin 2 receptor, gamma	18.1
20	IGHM	Immunoglobulin heavy constant mu	12.1	20	PVRIG	Poliovirus receptor rel. Ig domain cont	16.4
				21	***TSPAN7***	Tetraspanin 7, TALLA-1	16.1
**T-ALL VS. PBMC**							
1	TFRC	Transferrin receptor (p90, CD71)	21.2	1	***TSPAN7***	Tetraspanin 7, TALLA-1	55.0
2	***TSPAN7***	Tetraspanin 7, TALL-1	19.8	2	HHIP*	Hedgehog interacting protein	23.7
3	TBXA2R	Thromboxane A2 receptor	19.5	3	HMMR*	CD168 – RHAMM	12.8
4	CD3D*	CD3d molecule, delta (CD3-TCR complex)	19.4	4	IGSF10	Ig superfamily, member 10	11.4
5	FAF1	Fas (TNFRSF6) associated factor 1	16.4	5	VANGL1	VANGL planar cell polarity protein 1	6.8
6	HHIP*	Hedgehog interacting protein	14.6	6	TFRC	CD71-transferrin receptor	5.3
7	SLC39A8	SLC 39 (zinc transp.), 8	14.5	7	TRO	Trophinin	5.0
8	A1BG	Alpha-1-B glycoprotein	14.2	8	CACNB3	Ca++ channel, volt.-dep., beta 3 sub	4.3
9	OTOP2	Otopetrin 2	13.4	9	MAGED1	Melanoma antigen family D, 1	4.2
10	ITGAE	Integrin, alpha, CD103	13.3	10	CD3D*	CD3d molecule, delta (CD3-TCR complex)	4.1
11	TMEM237	Transmem. prot. 237 (tetraspanin)	12.9	11	CHRNA5	Cholinergic R, nicotinic, alpha 5	4.1
12	TRO	Trophinin	12.6	12	FAF1	Fas (TNFRSF6) associated factor 1	3.1
13	SLC22A7	SLC 22 (organic anion transporter), 7	12.5	13	PTK7	Protein tyrosine kinase 7	3.1
14	SLC7A5	CD98/amino acid transporter, light chain	12.2	14	A1BG	Alpha-1-B glycoprotein	2.8
15	CACFD1	Ca++ channel flower dom. cont. 1	11.8	15	GPC2	Glypican 2	2.4
16	IGH	Immunoglobulin heavy locus	11.1	16	LRRC37A3	Leucine-rich repeat containing 37, A3	2.2
17	TNFRSF21	TNF receptor superfamily, member 21	11	17	IGH	Immunoglobulin heavy locus	2.2
18	VANGL1	VANGL planar cell polarity protein 1	10.8	18	MRC2	Mannose receptor, C-type 2	2.1
19	FGFR1	Fibroblast growth factor receptor 1	10.6	19	SCNN1A	Na+ channel, non-volt.-gated 1 alpha sub	2.1
20	GPC2	Glypican 2	10.6	20	LGR4	Leucine-rich repeat cont. GPCR 4	1.9

*As for Table 2, T-ALL gene expression profiles were compared to either CD34 (+) HSC (top half of table) or PBMC (bottom half of table). Data are reported and ranked according to *T* score (left half of table) or fold-change (right half of table). Both Gene Symbol and Gene Title are given for each transcript. Those transcripts that are represented in Table 2 (where comparison was to an array of normal tissues) are followed by an asterisk in the Gene Symbol column. Those transcripts appearing in both the top and bottom half of the table are in bold, with gray shading*.

In a series of papers in the 1980s, Seon et al. described a unique human T-cell leukemia antigen identified by a monoclonal antibody, designated it TALLA (61, 62). Immunotoxin-conjugated antibody to TALLA was found to control the growth of leukemic xenografts in nude mouse models (63). The antigen, a tetraspanin, is now referred to as TSPAN7, is expressed from the X chromosome, and has been most recently been explored with regard to its role in differentiating glutamatergic neurons (64). As discussed above, little is known about HHIP and leukemia. As HHIP is likely to regulate stem cell-like characteristics of either leukemia cells, or stromal cells that support them it should be considered a high-value target (53). Also of interest was our detection of SLC7A5, which is also known as CD98, in the *T* score but not fold-change column of Table 3, where comparison is made to PBMC. A humanized monoclonal antibody to CD98, IGN523, is currently being tested in refractory or relapsed AML (NCT02040506). CD98 is composed of two chains, and has the ability to signal though both mTOR and AKT pathways (65).

All of the gene expression data are presented in our fully annotated Oncogenomics database^2^.

## Discussion

In seeking to analyze a series of pediatric malignancies for potential immunotherapeutic targets, we did not have a data set sufficiently robust to analyze pediatric T-ALL. In the data presented here, we now can add T-ALL to our list of pediatric malignancies that have been analyzed at the transcript level. Future studies will be enhanced by our ability to look at next-generation sequencing data, and to perhaps identify differential expression between tumor and normal at even finer levels, such as comparing alternative splicing profiles. Although we did not discuss our data for pre-B-ALL in a transcript by transcript level, our results were consistent with our earlier work looking at a more restricted set of normal tissues (4). The antigens CD19, CD79A, CD79B, CD49d, CD53, CD72, TLR1, and MILR1 were all reported previously. Thus, our pre-B-ALL analysis sets are consistent. New and potentially interesting hits that arose in this new analysis for B-ALL include CD52, CD24, and CD10/CALLA. Therapeutic antibodies have been developed for all of them.

The analysis of pediatric T-ALL is novel, and seems to reflect the perplexing nature of this disease in that the majority of the over-expressed transcripts are likely to be broadly expressed throughout the immune system. Thus CD2, CD69, and CD99 are all very risky targets that may require more sophisticated approaches than a single antibody or CAR-T. This holds true for CD96 as well, which is being used to purge bone marrow, but is too broadly expressed to be targeted systemically. We do have some reasonable antigens to target, but the data may be telling us something else. It may be time to consider more fully the use of clonotypic antibodies or targeted CAR-Ts to permanently eliminate specific T-cell subsets.

In the clinic, minimal residual disease can be detected in >95% of ALL patients by PCR detection of patient-specific rearrangements of the TCR or the immunoglobulin locus. This argues that at a genetic level the TCR is a valid target for therapeutic development (66). Campana et al. looked carefully at the expression of intracellular vs. membrane-expressed CD3 and of the TCR in T-ALL (67). In a series of ALL where patients ranged in age from 6 to 65 (median age, 20), 11 of 40 showed CD3 and either TCRAB (9 of 11) or TCRGD (2/11) on the membrane. Cytoplasmic TCR was detected in 12/40; while 17/40 had neither CD3 or TCR surface expression. In a study of adult T-ALL associated with HTLV-1 infection in Japan, all samples expressed surface TCR, albeit with a low fluorescence intensity (68). In this study, 7/12 patient samples expressed TCR on the surface on >95% of their blasts. Thus, the disease association with differentiation status holds, and we can state that a third of all patients with T-ALL are likely to have surface TCR. Unfortunately, our acuity in diagnosis of T-ALL according to the degree of cellular progression along the T-cell developmental pathway has not resulted in any useful differentiation of outcome status, with the possible exception of a slightly better outcome for CD10+ disease (69).

We propose a second method for targeting certain sub-types of T-ALL that express a surface T-cell receptor. We hypothesize that deletion of a specific family or sub-family of TCR-bearing ALL, for example the TCRBV5-4 family that ranked the highest of all differentially expressed genes in Table 2, would effectively eliminate that leukemic clone. Using CART-Ts specific for a single TCR family would result in only a partial depletion of normal T-cells, and the physiological consequences with respect to induced immunosuppression may be minimal. This is in contrast to current CD19–CAR-T therapy for B-ALL, where the entire healthy B-cell compartment is ablated. Alternatively, the generation of target lists, such as we present here may be exploited in the future when a new generation of immunotherapeutic agents where more than one set of antigens is required to fully activate an effector T-cell-can be developed. The creation of a treatment strategy for prostate cancer that requires recognition of both PMSA and PSCA to fully trigger a dual-engineered T-cell population may be one such approach (70). Our next step will be to validate surface expression of the transcripts described here by flow cytometry, and thus complement gene expression data with the demonstrated presence of protein that could be targeted on the surface of pediatric T-ALL.

Finally, we also explored the effect of altering the normal tissue comparator from a collection of normal tissues throughout the body, to two more restricted normal tissue sample sets. The first of these was the CD34+ HSC compartment in bone marrow. As this is the tissue from which the leukemia arises, one might anticipate that a more tumor-like set of transcript would be identified. However, the transcripts remained very much T lymphocyte specific, and up to 70% of the transcripts were shared when comparison was made between T-ALL and normal HSCs (Table 2 vs. Table 3). We did see a noticeably different set of transcripts identified when peripheral blood lymphocytes were used as the normal tissue comparison. The T lymphocyte specific transcripts were lost and a more generic tumor-like set of transcripts were noted. Interestingly, the CD3-delta chain of the TCR complex remained highly over-expressed in all comparisons, as did TALLA-1 (in 2 of 3) and HHIP (in 3 of 3). TALLA-1 has been described previously as a T-cell leukemia specific antigen and our analysis confirms that this is a high priority target worthy of further exploration (61). Shared between all comparisons was HHIP. HHIP has been elegantly described as a modulator of SHH signaling, adding another level of regulation to the patched (PTC1) and SMO pathway that modulates expression of Hedgehog (Hh)-regulated genes (52). A report demonstrating participation of this pathway in regulating myeloid leukemia supports further research into the role of HHIP in lymphocytic leukemia (53). In conclusion, there may be no single “correct” normal tissue to use as a comparator for gene expression profiles from leukemia samples. Instead, we should view each comparison as a means to identifying unique aspects of leukemia-associated gene expression patterns. These approaches highlight that general tissue comparisons yield results that reflect the tissue of origin of the malignancy, as seen in Table 2, while comparison to similar tissues types (in this case mature lymphocytes) reveals transcripts that are biased toward those that are associated with tumorigenesis, Table 3. However, both types of targets are of value, especially in the context of immunotherapy, where finding tumor-specific antigens that spare the host from fatal toxicity is the goal. If we restricted ourselves to the HSC or lymphocytic comparators in studying B-ALL, we would have missed antigens like CD19 and CD22 that are currently being targeted in experimental trials. When an antigen is found in all comparator scenarios, such as HHIP, it certainly warrants further investigation. This is also the case for TALLA-1, although it may not differ sufficiently from normal tissue. The antigens identified by using a broad tissue sample are certainly valuable, but as discussed, must be evaluated for the impact of targeting the normal tissues that may share these antigens, such as mature T-cell subsets.

In the Pediatric Cancer Genomes project, a database of prevalent mutations found in pediatric cancers, including T-ALL is being assembled^3^. Currently, this database is very useful for identifying mutations, which differs from our analysis, focusing on gene expression levels relative to normal tissue data sets. The largest collection of T-ALL samples at the Pediatric Cancer Genomes database is derived from ETP-ALL (9), and when this and other datasets are explored more closely it is apparent that only a very small minority of the genes identified are cell surface membrane proteins, with one exception, IL-7R. We also identified in IL-7R in T-ALL when HSC was used as a comparator in the fold-change analysis (Table 3), and as hit number 32 in the comparison run with our broad normal tissue panel (Table 2). This highlights again that different targets of interest will be identified when different normal comparators are used. This also encourages mining the “normal” T-cell developmental antigens we have identified for potentially harboring oncogenic mutations as well. The entire data in our fully annotated (including membranous protein assignment) gene expression database where users can search specific genes and compare gene expression between T-ALL, B-ALL, HSC, PBMC, and a range of normal tissue are available at: http://home.ccr.cancer.gov/oncology/oncogenomics/.

## Conflict of Interest Statement

The authors declare that the research was conducted in the absence of any commercial or financial relationships that could be construed as a potential conflict of interest.

## Footnotes

^1^http://www.arraymining.net

^2^http://home.ccr.cancer.gov/oncology/oncogenomics/

^3^http://www.pediatriccancergenomeproject.org/site/
